# Which is best for osteoporotic vertebral compression fractures: balloon kyphoplasty, percutaneous vertebroplasty or non-surgical treatment? A study protocol for a Bayesian network meta-analysis

**DOI:** 10.1136/bmjopen-2016-012937

**Published:** 2017-01-16

**Authors:** Shun-Li Kan, Zhi-Fang Yuan, Ling-Xiao Chen, Jing-Cheng Sun, Guang-Zhi Ning, Shi-Qing Feng

**Affiliations:** 1 Department of Orthopaedics, Tianjin Medical University General Hospital, Tianjin, China; 2 School of Nursing, Tianjin Medical University, Tianjin, China

**Keywords:** percutaneous vertebroplasty, balloon kyphoplasty, non-surgical treatment, osteoporotic vertebral compression fractures, network meta-analysis

## Abstract

**Introduction:**

Osteoporotic vertebral compression fractures (OVCFs) commonly cause both acute and chronic back pain, substantial spinal deformity, functional disability and decreased quality of life and increase the risk of future vertebral fractures and mortality. Percutaneous vertebroplasty (PVP), balloon kyphoplasty (BK) and non-surgical treatment (NST) are mostly used for the treatment of OVCFs. However, which treatment is preferred is unknown. The purpose of this study is to comprehensively review the literature and ascertain the relative efficacy and safety of BK, PVP and NST for patients with OVCFs using a Bayesian network meta-analysis.

**Methods and analysis:**

We will comprehensively search PubMed, EMBASE and the Cochrane Central Register of Controlled Trials, to include randomided controlled trials that compare BK, PVP or NST for treating OVCFs. The risk of bias for individual studies will be assessed according to the Cochrane Handbook. Bayesian network meta-analysis will be performed to compare the efficacy and safety of BK, PVP and NST. The quality of evidence will be evaluated by GRADE.

**Ethics and dissemination:**

Ethical approval and patient consent are not required since this study is a meta-analysis based on published studies. The results of this network meta-analysis will be submitted to a peer-reviewed journal for publication.

**PROSPERO registration number:**

CRD42016039452; Pre-results.

**Strengths and limitations of this study**

- This is the most comprehensive review comparing the efficacy and safety of percutaneous vertebroplasty, balloon kyphoplasty and non-surgical treatment for patients with osteoporotic vertebral compression fractures through a Bayesian network meta-analysis.
- We will use the Grading of Recommendations Assessment, Development and Evaluation (GRADE) approach to evaluate the quality of evidence.
- The results of this study will help surgeons and patients to select appropriate treatments.
- This study is based on the quantity and quality of the trials available for review.

## Introduction

Osteoporosis is a systemic bone disorder with reduced bone mass and degradation of skeletal microarchitecture.^1^ Patients with osteoporosis frequently experience vertebral compression fractures.^2^ Vertebral compression fractures usually happen after a disruption in the vertebral column, particularly the collapse of the front of the vertebrae. Osteoporotic vertebral compression fractures (OVCFs) constitute a major health problem, because they commonly cause both acute and chronic back pain, substantial spinal deformity, functional disability, decreased quality of life and high treatment expenses.^3^ ^4^ It is reported that the incidence of OVCFs in individuals aged 50 years or older is 307 per 100 000 person year and the direct expenses concomitant with a new onset of OVCFs in the first year are ∼€6490.^5^ They also increase the risk of future vertebral fractures and mortality.^6^

The purposes of treatment for OVCFs are to restore mobility, reduce pain and avoid new fractures.^7–9^ Non-surgical treatment (NST) includes bed rest, various pharmacological agents, back braces and physical therapy, which are used to relieve symptoms and strengthen the spine.^10^ ^11^ Although most vertebral compression fractures gradually cure in a few months, some patients have lasting pain and disability, and require invasive intervention.^12^ Percutaneous vertebroplasty (PVP) was first introduced in 1987 as a new intervention to treat vertebral angioma.^13^ Until now, it has been widely used in patients suffering from OVCFs and concomitant back pain.^14^ Balloon kyphoplasty (BK) is a relatively novel technique which alleviates pain and improves function by using inflatable bone tamp to compress the cancellous bone.^15^ ^16^

These treatments are widely used. However, which treatment is preferred is unknown. More knowledge regarding the efficacy and safety of these treatments, which would be beneficial to patients, surgeons and policymakers, is therefore required to establish reasonable treatment hierarchies for OVCFs. The relative efficacy and safety of OVCF treatments are difficult to identify from previous pairwise meta-analyses, because head-to-head comparison studies are not available for all of the treatments and traditional pairwise meta-analyses cannot pool all of the evidence from several interventions, while decision-makers need to know the relative ranking of a set of alternative treatments and not just whether option A is better than option B. Moreover, although lots of traditional pairwise meta-analyses comparing these treatments for OVCFs exist, most of them compare PVP with NST and few meta-analyses contrasted BK with PVP or NST. When no studies exist that directly compare all relevant treatment choices, a network meta-analysis can be performed by comparing the relative effects of treatments against a common comparator or combining a variety of comparisons that are taken together from one or more chains linking the treatments of interest.^17^ Consequently, it is necessary to perform a network meta-analysis to ascertain the relative efficacy and safety of BK, PVP and NST for patients with OVCFs. Although Chen *et al*^18^ compared the efficacy and tolerability of these treatments for OVCFs in old people, only five studies with 777 participants were included.

Therefore, the purpose of this study is to comprehensively review the literature and ascertain the relative efficacy and safety of BK, PVP and NST for patients with OVCFs using a Bayesian network meta-analysis.

## Methods

### Design

A network meta-analysis based on a Bayesian framework will be performed in this study.

This protocol of network meta-analysis will be carried out according to the Preferred Reporting Items for Systematic review and Meta-Analysis Protocol (PRISMA-P),^19^ and the PRISMA extension statement for reporting of systematic reviews incorporating a network meta-analysis of healthcare interventions.^20^ This study has been registered at PROSPERO (http://www.crd.york.ac.uk/PROSPERO) with registration number CRD42016039452.

### Eligibility criteria

1. Type of study

All relevant randomised controlled trials will be included. Quasi-randomised controlled clinical trials, with method of allocation by alternation or date of birth, will not be included. The language or date of publication will not be limited. Only studies with full texts will be considered for inclusion.

1. Participants

Trials enrolling adults, aged at least 18 years, with a diagnosis of OVCFs of any duration will be included. Studies with vertebral compression fractures caused by major trauma or malignancy will not be included.

1. Type of interventions

We will include trials to assess the effectiveness and safety of PVP, BK and NST in patients with OVCFs. PVP is defined as percutaneous injection of bone cement into a vertebral body under imaging guidance. BK is analogous to PVP, but a balloon is inserted into the vertebral body and expanded before bone cement is injected. NST consists of sham procedure, pharmacological treatment, bracing, physical therapy and usual care (best supportive care).

1. Outcomes of interest

The efficacy outcome will be pain measured on the visual analogue scale or numeric rating scale, a scale from 0 to 10, in which 0 denotes no pain and 10 denotes the maximum level of pain; the European Quality of Life–Five Dimensions (EQ–5D) scale (scores range from 0 to 1, with 1 indicating perfect health); the score on a modified 23-item version of the Roland-Morris Disability Questionnaire (RMDQ, in which scores range from 0 to 23, with higher numbers indicating greater physical disability); the Oswestry Disability Index (ODI); and scores on the physical component summary and mental component summary subscales of the self-administered Medical Outcomes Study 36-Item Short-Form General Health Survey (SF-36).

The outcome regarding safety will be subsequent vertebral fractures, which consist of symptomatic vertebral fractures and radiographic vertebral fractures; refractures at treated levels; and adjacent level fractures.

We will choose the longest follow-up time as the measurement time point for all of the outcomes.

### Data sources and search strategy

We will comprehensively search PubMed, EMBASE and the Cochrane Central Register of Controlled Trials (CENTRAL) from their inception to June 2016. Language or publication period will not be limited. The search strategy will include related text words and medical subject headings regarding OVCFs, BK, PVP, NST and randomised controlled trials. The details of the search strategies are shown in online supplementary file S1. Additionally, we will also search *ClinicalTrials.gov*, the Australian New Zealand Clinical Trials Registry (ANZCTR) and the Chinese Clinical Trial Register (ChiCTR) for ongoing trial registers and verify related reports on the US. Food and Drug Administration website. Related systematic reviews and meta-analyses through initial retrieval will be checked and bibliographies will be dissected for additional-related studies.

10.1136/bmjopen-2016-012937.supp1 supplementary data

### Study selection

Titles and abstracts of the identified searches will be independently screened by two investigators (S-LK and Z-FY). Trials that do not meet the eligibility criteria will be excluded. After omitting the duplicated and obviously unrelated studies, we will review all of the remaining studies in full text and ascertain whether they should be included by the same eligibility criteria. Any discrepancies will be solved by a third reviewer. When multiple publications are from the same data set, the study with the most complete data and the longest follow-up will be included. Excluded studies and the reasons for exclusion of them will be reported and confirmed by a third investigator.

### Data extraction

Two investigators (S-LK and Z-FY) will independently extract the information from the original studies using a standardised data abstraction list, including study characteristics (such as first author, publication year, country and sponsor), patient characteristics (such as number of patients, mean age, gender ratio, the duration of symptoms and inclusion/exclusion criteria), intervention details for each treatment group (eg, intervention type, the type of anaesthesia, the duration of follow-up and cointerventions) and outcome measures (pain, EQ–5D, RMDQ, ODI, SF-36, subsequent vertebral fractures, refractures at treated levels and adjacent level fractures). Numerical data will be abstracted to compute pooled estimates. Data that could not be got from the texts directly will be recalculated, if possible. If both final values and change from baseline values for the same outcome are reported in a study, we will select the measure that is frequently measured. Any disagreements will be resolved by consensus among all the investigators.

### Risk of bias assessment

Two independent investigators (S-LK and Z-FY) will appraise the risk of bias for individual studies according to the Cochrane Handbook.^21^ The criteria for assessment involve randomisation, allocation concealment, blinding of participants and personnel, blinding of outcome assessors, incomplete outcome data, selective reporting and other biases. Each of the domains is determined as ‘low risk’, ‘unclear risk’ or ‘high risk’. Studies with a high risk of bias in one or more key items will be regarded to be at a high risk of bias. Studies with a low risk of bias in all key items will be regarded to be at a low risk of bias. Otherwise, they will be regarded to be at an unclear risk of bias.^22^ Disagreements will be resolved via a discussion with a third author.

### Statistical analysis

First, we will do a traditional pairwise meta-analysis, which is used for consistency check and an evaluation of heterogeneity, for all available direct evidence comparing two treatments using Stata, V.13.0 (Stata Corp, College Station, Texas, USA). The I^2^ statistic will be applied to quantify the extent of between-trial heterogeneity, with I^2^ >50% indicating considerable heterogeneity. The random-effects model will be used as the main model. Furthermore, the results of the random-effects model will be compared with that of the fixed-effects model to test the stability of the results. OR with 95% CI will be calculated for a dichotomous variable. Mean difference (MD) with 95% CI will be estimated for a continuous outcome.

Network meta-analysis will be conducted using a Bayesian Markov chain Monte Carlo (MCMC) framework and fitted in R V.3.2.4 software (https://cran.r-project.org/src/base/R-3/) via the gemtc V.0.81 package. A Gaussian model will be used for the continuous variables and a Bernoulli model will be used for the dichotomous variables. The posterior distribution of the parameter which is used for inference will be summarised by its median (OR or MD) and 95% credible interval (CrI). Three chains with different initial values will be run simultaneously. For the analyses, inference will be based on 150 000 iterations of MCMC after a 50 000 iteration burn-in period.^23^ To assess convergence, trace plots and Brooks-Gelman-Rubin diagnostic plots will be used.^24^

To check whether a model's fit is satisfactory, the posterior mean residual deviance, an absolute measure of fit, will be calculated. Then we will compare the value of posterior mean residual deviance with the number of independent data points to test if the model fits the data well.^25^ We will compare fixed-effect and random-effect models using the deviance information criterion (DIC), which is a measure of model fit that penalises model complexity. The model with lower DIC values will be adopted, with differences of five or more units regarded as meaningful.^26^ If a similar DIC emerges in the two models, a fixed-effect model will be adopted; however, if there is significant heterogeneity in the pairwise comparison, a random-effect model will be used. Consistency will be evaluated by comparing the model fit from a consistency model with that from an unrelated mean effect model^27^ and by comparing direct evidence from pairwise meta-analysis with indirect evidence using the node-split approach.^28^

Clinical and methodological heterogeneity will be assessed through examining the characteristics and design of included studies. Statistical heterogeneity among the studies and in the entire network will be evaluated based on the magnitude of heterogeneity parameter (I^2^ or τ^2^) estimated from network meta-analysis models.^29^ I^2^ >50% denotes considerable heterogeneity. Heterogeneity will be investigated by fitting covariates (ie, mean age, female ratio, sample size, the duration of symptoms and the duration of follow-up) in network meta-regression.^30^ Subgroup meta-analyses will be further performed based on the duration of symptoms (acute (≤6 weeks), subacute (6–12 weeks) or long-standing (>12 weeks)) and the duration of follow-up (short term (<1 year) or long term (≥1 year)), if possible. Sensitivity analyses will be performed to examine the robustness of the outcomes by excluding studies at high risk of bias assessed by the Cochrane risk of bias tool, excluding studies with follow-up <6 months, excluding studies in which any comparator group consists of fewer than 50 participants and restricting analyses to studies with adequately concealed allocation.

For each outcome, we will assess the probability that each treatment regimen is the best (superior to all other interventions), second best, third best, etc, according to the rank order of the treatment regimens in each iteration of the Markov chain. Then the surface under the cumulative ranking (SUCRA), a summary of the probabilities that each intervention arm is associated with being the most effective, will be calculated. A treatment with a SUCRA value of 100 is certain to be the best, whereas a treatment with 0 is certain to be the worst.^31^

To visually assess the presence of small-study effects in the network, comparison-adjusted funnel plots will be applied.^32^ Funnel plots in a network meta-analysis account for the fact that studies evaluate treatment effects for different comparisons. For the comparison-adjusted funnel plot, the horizontal axis will represent the difference between study-specific effect sizes from the comparison-specific summary effect. In the absence of small-study effects, the comparison-adjusted funnel plot should be symmetric around the zero line.

### Quality of evidence

The quality of evidence will be evaluated following the Grading of Recommendations, Assessment, Development and Evaluation (GRADE) four-step method for grading the quality of treatment effect estimations from this network meta-analysis:^33^ (1) Present direct and indirect treatment estimates for each comparison of the evidence network. (2) Rate the quality of each direct and indirect effect estimate. (3) Present the network meta-analysis estimate for each comparison of the evidence network. (4) Rate the quality of each network meta-analysis effect estimate. On the basis of parameters such as risk of bias, inconsistency, indirectness, imprecision and publication bias, the quality of evidence will be rated as high, moderate, low or very low. GRADE pro, V.3.6 (http://www.gradeworkinggroup.org/) will be applied to perform this process.

## Ethics and dissemination

### Ethical issues

Since no primary data collection will be undertaken, no additional formal ethical assessment and no informed consent are required.

### Publication plan

This network meta-analysis will be submitted to a peer-reviewed journal. It will be disseminated electronically and in print.
