# Supplementary material for: Which is best for osteoporotic vertebral compression fractures: balloon kyphoplasty, percutaneous vertebroplasty or non-surgical treatment? A study protocol for a Bayesian network meta-analysis
Source: BMJ Open. 2017 Jan 16;7(1):e012937. doi: 10.1136/bmjopen-2016-012937 (PMC5253565; doi:10.1136/bmjopen-2016-012937)
Supplement: supplementary data [file bmjopen-2016-012937supp.pdf]

PubMed:

#1	"Randomized Controlled Trial" [Publication Type] OR "Controlled Clinical Trial" [Publication Type]
#2	((((placebo[Title/Abstract]) OR randomized[Title/Abstract]) OR randomly[Title/Abstract]) OR trial[Title/Abstract]) OR groups[Title/Abstract]
#3	("Animals"[Mesh]) NOT "Humans"[Mesh]
#4	(#1 OR #2) NOT #3
#5	"Spinal Fractures"[Mesh]
#6	"Fractures, Compression"[Mesh]
#7	#5 AND #6
#8	(spinal compression fracture[Title/Abstract]) OR vertebral compression fracture[Title/Abstract]
#9	#7 OR #8
#10	"Kyphoplasty"[Mesh]
#11	"Vertebroplasty"[Mesh]
#12	((((((((((((((percutaneous vertebroplast*[Title/Abstract]) OR pereutancous vertebroplast*[Title/Abstract]) OR Vertebroplast*[Title/Abstract]) OR Cementoplast*[Title/Abstract]) OR Sacroplast*[Title/Abstract]) OR balloon kyphoplast*[Title/Abstract]) OR kyphoplast*[Title/Abstract]) OR conservative treatment*[Title/Abstract]) OR optimum pain treatment[Title/Abstract]) OR optimal medical therap*[Title/Abstract]) OR nonsurgical therap*[Title/Abstract]) OR Nonsurgical

treatment[Title/Abstract]) OR non-surgical therap*[Title/Abstract]) OR Non-surgical treatment[Title/Abstract]) OR Non-surgical management[Title/Abstract]) OR Nonsurgical management[Title/Abstract]

#13 #10 OR #11 OR #12

#14 #4 AND #9 AND #13
